# Can a single pollen measurement site provide exposure information for health research across an entire state? Results from a study of allergic-type asthma associated with thunderstorms (2007–2018)

**DOI:** 10.1038/s41370-025-00777-z

**Published:** 2025-05-05

**Authors:** M. Luke Smith, Richard F. MacLehose, Jesse D. Berman

**Affiliations:** 1 Division of Epidemiology and Community Health, University of Minnesota School of Public Health, Minneapolis, MN 55455 USA; 2 Social Science Research Institute, The Pennsylvania State University, University Park, PA 55455 USA; 3 Division of Environmental Health Sciences, University of Minnesota School of Public Health, Minneapolis, MN 55455 USA

**Keywords:** Air Pollution, Children’s Health, Health Studies, Exposure assessment

## Abstract

**Background:**

Thunderstorm asthma is an increase in severe asthma following thunderstorm events during high pollen conditions. However, sparse pollen measurements hinder epidemiological research of this phenomenon.

**Objective:**

Is pollen measured at a single site predictive of thunderstorm asthma risk across a broad region?

**Methods:**

We conducted a meta-analysis to estimate thunderstorm asthma risk on 19 city-level sites incorporating local weather and patient data but a single pollen site. We use meta-regression to explore effect modification by land cover and distance from pollen measurement location.

**Results:**

Meta-analysis showed no evidence of a state-wide thunderstorm asthma effect. Meta-regressions suggest that increased vegetation was associated with higher thunderstorm asthma risk with reduced risk at greater distances from pollen collection sites.

**Impact statement:**

The phenomenon of thunderstorm asthma in the U.S. remains poorly studied due to geographically sparse pollen collection sites. Using a 19-city study, we demonstrate that incorporating environmental characteristics, such as land cover of allergic-type pollen-producing grasslands and deciduous trees, can improve the prediction of thunderstorm asthma risk at far distances from pollen monitors. By increasing the precision of pollen estimates, we can improve the estimation of thunderstorm asthma human health risks and potentially optimize decisions for new pollen collection sites.

## Background

Thunderstorm asthma occurs when pollen particles break down during thunderstorm conditions, releasing respirable sub-particles that can trigger asthma [1, 2]. Officially recorded less than 30 times [3], thunderstorm asthma has been mainly marked by severe epidemic events [4–6]. In recent work, we reported the presence of non-epidemic thunderstorm asthma occurring at a lower magnitude with a general increase of 1.05 times higher risk of asthma emergency department (ED) visits following thunderstorms occurring with elevated pollen concentrations [7].

Investigating thunderstorm asthma in the U.S. is challenged by sparse measurement of pollen data, with fewer than 60 stations available nationwide [8]. Previous research has demonstrated that pollen levels show evidence of homogeneity up to distances of 30 km [9]. Yet questions persist regarding the suitability of sparse pollen stations to evaluate thunderstorm asthma events across large distances and if other data can be used as a pollen surrogate. Pollen seasons are driven by a combination of seasonal photoperiod plus daily temperature and local weather [10], so it is possible that similar pollen levels may be present across a larger area at an identical time of year with variation attributed to local weather.

We seek to determine if pollen measured at a single site has face validity for estimating thunderstorm asthma risk across a broad geography of 19 sites in Minnesota, U.S.A [11]. We will evaluate if the effect of thunderstorm asthma events on ED visits varies with distance from a single monitoring site [11], and the role of landcover type as a proxy for pollen species in different parts of the state. This preliminary study will inform the distance at which pollen values can be extrapolated for exposure research related to thunderstorm asthma and support the evaluation of national risks, which have not been evaluated.

## Methods

### Study population and health data

From the 24 largest U.S. Census designated core-based statistical areas (CBSA) in Minnesota [12] with urban centers of at least 10,000 (eFig. 1), we eliminated 3 locations because of overlapping boundaries and 2 for lack of data. The 19 remaining sites encompassed all zip code populations wholly or partially within 15 miles of the urban center of the CBSA [13], or the Minneapolis-St. Paul (MSP) airport for the Minneapolis/St. Paul/Bloomington metropolitan area. Daily counts of asthma-related emergency department (ED) admissions from 2007 to 2018 with first or second diagnosis code (ICD-9 493, ICD-10 J45) [14] for all study area zip codes were extracted from Minnesota Hospital Association Uniform Billing Claims Data accessed under agreement with the Health Economics Program of the Minnesota Department of Health (MDH) [15]. These records account for 96% of hospital bed discharge records in the state of Minnesota [15].

### Thunderstorm asthma events

Thunderstorm asthma events were defined as two or more lightning strikes in a day occurring in the presence of high or very high pollen (> 75^th^ percentile) [16]. We assign lightning exposure using a 0.1-degree grid of daily lightning counts from the VAISALA lightning detection network and using inverse distance weighting [17] calculate the weighted daily value at each study area’s centroid. National Allergy Bureau pollen data for Minnesota for a single site in Minneapolis near the airport is counted by certified pollen counters employed by the Clinical Research Institute of Minneapolis, which provided the data [18]. This is the only pollen measurement site in Minnesota.

### Environmental data

Landcover categories were obtained from the 2013 Minnesota Landcover Classification at a 15 m resolution [19]. (eFig. 2) Using GIS tools, we calculated the total area of ten unique land cover types inside each 15-mile radius study area. Only land cover within the 15-mile radius was included to keep exposure areas aligned across study sites. Daily weather measurements from Automated Surface Observation Stations (total precipitation, maximum wind speed, maximum relative humidity, and maximum temperature) were downloaded from the Iowa Mesonet [20], and air pollution data for average ozone and maximum fine particulate matter (PM_2.5_) from the Environmental Protection Agency [21]. All weather and pollutant values at each site were interpolated to communities using nearest neighbor or inverse distance [16].

### Statistical analysis

In our two-stage approach, we first ran individual quasi-Poisson regression models with day as the unit of analysis for the 2007–2018 study period from April to October and calculated the rate ratio of asthma ED visits associated with thunderstorm asthma events adjusted for all covariates, with an annual population offset, and a 6-knot cubic spline term to account for seasonality of asthma. We fit models for effect on the day of the thunderstorm asthma event and the subsequent day (lag-1). Missing values for pollen data are estimated for up to three consecutive missing days as the mean of the days that bound the missing period [16]. Sensitivity tests were conducted with 5 and 7 degrees of freedom for the spline term. In a second stage, we combined the individual study area estimates using random effects meta-analysis to calculate an overall risk [22]. To investigate demographic differences, this analysis was repeated for subgroups, created based on age and sex, with analyses for the entire population, male, female, under age 18, 18-44, and 45 and up, and age-sex categories for male and female under 18, male and females 18-44, and male and female 45 and up. Categories were chosen to ensure adequate cell count, to identify children at risk, and to match other literature about severe asthma risks by age group. Details of exposure assignment, model specification, and effects of covariates are described in the eSupplement and in previously published work [7].

Following our meta-analysis, we fit a series of meta-regressions to investigate explanatory variables for between-site effects and investigate the ability to improve estimations over a large study area where pollen measures are absent through landcover surrogates. Our covariates included plant cover type, an overall landcover metric indicative of pollen, and measures of absolute distance from each site from the true pollen measurement location.

To estimate the effect of plant cover types, we calculated the area and percent coverage of different plant types in our study areas for land categories of conifer and deciduous forest, emergent wetland, extraction, forested and shrub wetland, hay and pasture, managed grass/natural grass, mixed forest, open water, and impervious (urban). To approximate pollen exposure associated with the grass, tree, and weed pollen of health concerns, we created a composite value based on the square miles of combined deciduous tree or managed grass/natural grass in each study region. Prior research indicates that grass, ragweed, birch, elm, hazel, and alder trees have all been associated with the distribution of sub-pollen particles in the specific conditions of a thunderstorm [23]. We calculated the distance from the centroid of each CBSA study unit to MSP airport, our proxy for our anonymized pollen collection center. We fit meta-regression models using these measures individually and, as a sensitivity analysis, re-ran the meta-regressions excluding Minneapolis–St. Paul, the largest metro area. To test whether distance and land cover are associated, we measured the correlation between distance and square miles of deciduous trees or managed or natural grassland. We also fit meta-regressions for square miles and percentage coverage of our pollen proxy landcover and distance from Minneapolis St. Paul, excluding the six sites clipped by the state border.

In further sensitivity analyses, we explored the robustness of our thunderstorm asthma definition by using alternative exposures of (1) pollen only (high/very high pollen in the absence of lightning) and (2) lightning only (with low pollen <25%). Second, we ran separate meta-regressions to test whether heterogeneity between lightning-only and asthma was modified by distance from MSP or area of land cover hypothesized to contain involved pollen types. Third, we repeated this process for subgroups by age and sex using a meta-analysis of models with high pollen only and meta-regression using square miles of land cover with potential for asthma-producing pollen types and for distance from the MSP pollen collecting station. Fourth, we considered the effect of square miles of ‘all other land types’ as a counter to our meta-regression term of wild or managed grass and deciduous trees. Fifth, we tested a one-at-a-time addition of single landcover types to our pre-defined deciduous plus grass landcover measure.

## Results

Table 1 shows the 19 non-overlapping Minnesota urban study areas included in the analysis, their 2010 overall population, mean annual emergency department visits for asthma (with SD) and mean annual storm events (with SD) from 2007 to 2018.

**Table 1:** Descriptive statistics on population, emergency department cases of severe asthma and location-specific storms occurring during high Minneapolis pollen counts for 19 urban locations.

Name	2010 Population	Mean Asthma Cases/ year	SD	Storms/year	SD	Mean Cases/ 10,000 person-year
Minneapolis-St. Paul	2281807	11959.6	928.8	9.18	3.63	52.4
Albert Lea	69,783	371.5	119.8	12.36	4.80	53.2
Alexandria	63,667	132.2	23.3	8.73	5.62	20.8
Bemidji	45,449	269.6	78.8	6.45	3.39	59.3
Brainerd	71,631	315.9	49.4	8.36	3.72	44.1
Duluth	145,842	811	75.4	7.27	3.04	55.6
East Grand Forks	24,443	89.8	17.2	7.00	2.86	36.7
Faribault	125,727	442.7	57.5	12.36	4.18	35.2
Fergus Falls	42,810	126.6	20.5	9.09	4.25	29.6
Grand Rapids	41,920	279.4	94.5	7.09	4.35	66.7
Hutchinson	66,784	191.3	43.6	11.73	4.00	28.6
Mankato	130,977	404.5	74.9	11.27	4.90	30.9
Marshall	35,034	65.9	11.8	9.27	3.88	18.8
Red Wing	75,686	300	113.2	11.45	3.72	39.6
Rochester	179,366	595	139.5	11.27	3.72	33.2
St. Cloud	194,217	1369.9	158.7	9.64	3.96	70.5
Willmar	48,662	107.5	13.9	10.55	5.16	22.1
Winona	59,265	126.3	25.6	11.27	4.38	21.3
Worthington	26,881	48.9	11.8	12.18	4.64	18.2

**Fig. 1:**
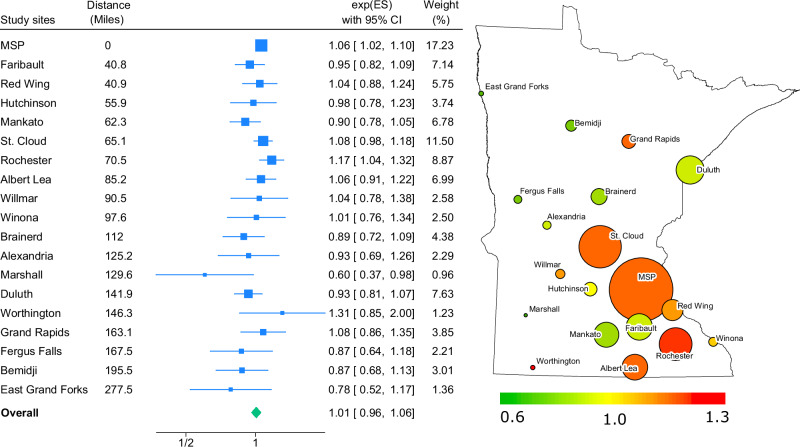
Meta-analysis of relative risk estimates (95% CI and DerSimonian&Laird weight) for ED asthma visits on ‘thunderstorm asthma’ days and map of 19 CBSA locations in Minnesota. Color denotes relative risk estimates, and size indicates weight. Sorted by distance to Minneapolis-St. Paul (MSP).

Meta-analysis of the 19 regression results across the largest population centers in Minnesota showed no evidence of a combined thunderstorm asthma effect (RR = 1.01; 95% CI = 0.96,1.06) and evidence of moderate heterogeneity of the effect (Heterogeneity I^2^ = 36.14%). These results are shown in Fig. 1, along with a map showing the effect estimate and study weight for each study region. The meta-analysis by age-sex subgroups indicated that there may be evidence of a combined effect for males 18–45 (RR = 1.10; 95% CI = 1.03, 1.18) and females 18–45 (RR = 1.08; 95% CI = 1.03, 1.13), with no evidence for other subgroups. These results are shown in the eSupplement.

**Fig. 2:**
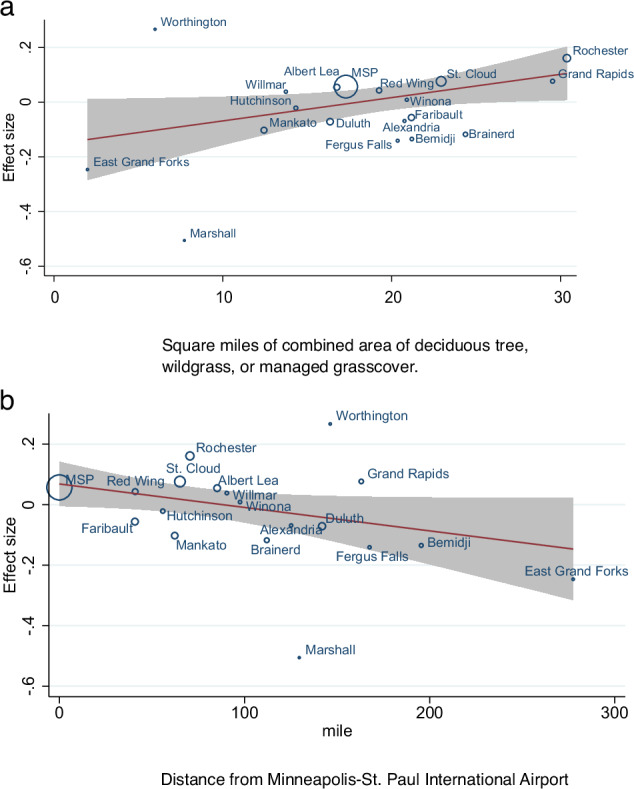
Meta-regression bubble plots. Change in relative risk of thunderstorm asthma associated with (**a**) total square miles of deciduous tree, wildgrass, or managed grass land types and (**b**) distance from Minneapolis-St. Paul airport.

To explore causes of effect heterogeneity across our 19 CBSA regions, our meta-regression using total square miles of plant types associated with prior thunderstorm asthma research (deciduous trees, grasses) found that each 10 mi^2^ area of ‘deciduous tree’ or ‘grass, managed or wild’ in zip codes within 15 miles of each CBSA study centroid, there was a 1.009 times higher effect size of relative risk (95% CI = 1.001, 1.017) (Fig. 2a). This relationship does not change when Minneapolis-St. Paul is excluded from the analysis. Similarly, if the percentage of land cover type for each study is used as the predictor, we find that for each one percent increase of the area within each study covered by relevant plant types, there is a 1.003 times higher effect size of severe asthma (0.998, 1.008), with similar results if Minneapolis-St. Paul is excluded (1.004 times higher effect size; 95% CI = 1.000, 1.009). Alternatively, we found the asthma effect was reduced with distance as each additional 10 mi distance from the pollen collection site is associated with a reduced relative risk (RR = 0.992 (95% CI = 0.984, 1.000)) (Fig. 2b). Restricting the meta-regression to study sites entirely within the state boundary does not change the estimate of effect measure modification for every 10 miles of distance from MSP (RR = 0.992 (95% CI = 0.981, 1.003)) or each additional 10 square miles of deciduous tree/grassland landcover (RR = 1.011 (95% CI = 1.000, 1.021)). Meta-regressions for the effects of landcover type and distance on age-sex subgroups showed similar patterns and are depicted in the eSupplement.

We found no evidence of correlation between square miles of tree plus wild or natural grass landcover type and distance from the MSP pollen measuring site (rho (df=17) = –0.315, *p* = 0.189).

Meta-analysis with alternate exposure of lightning only (2+ strikes) in the presence of low pollen shows no evidence of association (RR = 1.01 (95% CI = 0.96, 1.07)), with no evidence of heterogeneity (I^2^ of 6.7%), indicating that storms without pollen do not increase asthma hospitalizations. Meta-regression of this analysis shows no evidence of effect modification by square miles of pollen type (RR = 1.003 (95% CI = 0.993, 1.014)) and no evidence of effect modification by distance from MSP (RR = 1.000 (95% CI = 0.999, 1.001)).

When we conducted meta-analysis during high pollen only and no thunderstorm occurrence, we observed modest evidence of a protective association (RR = 0.98, 95% CI = 0.96, 1.00) with no evidence of heterogeneity (I^2^ = 3.4%), and meta-regression using 10 square miles of potentially asthma-producing pollen types shows a weakly protective effect (RR = 0.994, 95% CI = 0.990, 0.998). Meta-analysis of distance from the MSP pollen collecting station shows no evidence of an association between distance and change in effect size (RR = 1.000, 95% CI = 0.999, 1.001).

When we tested the effect of each 10 square miles of ‘all other land types’ as a counter to our meta-regression term of wild or managed grass and deciduous trees, we found no evidence of an association with the effect size of asthma relative risk (RR = 1.0, 95% CI = 0.996, 1.004). Adding pasture to our pre-defined deciduous plus grass landcover measure showed similar overall meta-regression values per 10 square miles (RR = 1.007; 95% CI = 1.000, 1.014), as did emergent wetland, a combination of weed and deciduous trees in wetland, (RR = 1.009; 95% CI = 1.001, 1.017). Other combinations had lower predictive value as a potential effect modifier.

## Discussion

Empirical research has found that distances ranging from 25 to 41 km have a good correlation of pollen measures [9, 24, 25]. In the United States, consistent pollen collection data stations are much sparser, with approximately 85 stations nationwide [26]. Using the information from the single pollen site available in Minnesota, our findings indicate that for evaluating thunderstorm asthma events, there is some predictive value of pollen levels at distances greater than previously tested. However, the measured effect diminishes with increasing distance. This reduced effect could be caused by misclassification or some other unmeasured factor. We found that the combined area of deciduous trees plus grassland is positively associated with the effect size of the relative risk of severe asthma on the day of thunderstorm events in 19 study locations, suggesting effect modification.

Our findings suggest that locations with plants similar to a remote pollen measurement location may have comparable daily pollen loads and provide predictive value for thunderstorm asthma research. In contrast, the linear decreasing effect size associated with greater distance from the central pollen site reflects an expected exposure measurement error that would plausibly lead to a biasing towards the null of health estimates at increasing distances [9, 24, 25].

Our a priori selection of deciduous trees and grassland land cover types is plausible based on previous literature demonstrating that plants, including ryegrass, or some tree species such as birch, olive, and grassland weeds, including pellitory [23], may have some involvement. However, this vegetation choice also lacks precision given the absence of additional species information and, more importantly, the nascence of the literature on pollen types associated with allergic-type thunderstorm asthma. Advancing the epidemiology of thunderstorm asthma requires a deeper understanding of what vegetative species most contribute to sub-pollen particle loads and how this can differ by time of year and geographic location. Limitations:

This study has several limitations. Exposure is ecological, and we cannot know the true exposure of any individual. Additionally, we cannot know if travel times to emergency departments affect utilization for less severe cases; however, all sites have a centrally located emergency department, and the population-weighted mean distance to an emergency room for each resident is 4.6 miles (2.0, 7.7) [27]. While we have explored differences in risk by age and sex during thunderstorm asthma, the data provided by the Minnesota Department of Health contains no information about race, ethnicity, or other social or demographic factors, and we could not examine their potential association with exposure or disease. Landcover data is a crude approximation of true pollen types. With only one single-point measurement for pollen, we cannot directly examine the predictive capacity of our measurement. We used a 15-mile radius area for each site for landcover measurement, but because of state boundaries, six sites are clipped and have smaller areas. This makes comparisons based on area percentages difficult as denominators differ and potentially undercount the total amount of square miles of land cover exposure. However, this approach best aligns with our health data as that is also not available for zip codes in neighboring states. We use a single year of landcover data to estimate landcover type, which could introduce additional bias or measurement error. Severe asthma is relatively rare, as are thunderstorm asthma events, which could introduce errors due to chance or lead to imprecise measurement. While our individual site models include ozone and PM_2.5,_ monitoring sites, these are not equally distributed throughout the state, which could introduce confounding; however, prior research found no evidence that these variables were associated with the outcome [16]. The phenomenon of thunderstorm asthma is poorly understood, and there is much to be explored about the factors that contribute to thunderstorm asthma events, including exploring effects by individual plant species, and further exploration of how meteorologic factors such as rain, wind speed and storm type, may affect the process of sub-pollen particle creation and dispersal and modify the association between storms, pollen and asthma [28].

Conclusions Improved estimation of pollen for health research requires accurate modeling and measurement for exposure purposes. Current commercial pollen models are based on historical data and current weather [29, 30] and some incorporation of species-specific landcover data can improve the underlying data for prediction [31]. However, at this time, many pollen prediction models report poor correlation with ground-measured local pollen counts [32], and while computationally intensive models are improving, their results remain similar to commercial models [33].

Future researcher might combine vegetation landcover data [34] with NDVI (normalized difference vegetation index) to test whether pollen seasonality is indeed evidenced. Another approach that would provide greater precision in pollen estimation would be to add additional pollen collection stations. Current developments in automated pollen collection systems may support this in the near future [35], however, at this time, automated pollen measurement may allow faster counts but does not provide counts as accurate as more labor-intensive manual methods [36]. Our research suggests additional pollen measurements might allow for more accurate thunderstorm asthma prediction, and previous work suggests that a greater density of pollen measurements is required [9, 24, 25]. It is plausible that improvements in spatiotemporal modeling of daily pollen loads, similar to advances in air pollution exposure assessment [37, 38], will be a future data source for public health research. However, while our results suggest that land cover can improve prediction, improved modeling and forecasting is currently limited by the limited number of pollen stations with standardized data, imprecise data on land cover and plant speciation, as well as a need for more research investment to develop better informed models that account for regional variation in weather or climatic regimes [39].

Current automated systems that measure pollen show 84% concordance with ‘gold-standard’ human analysis [25, 40], allow faster reporting times with lower labor costs. Allergic rhinitis is costly, associated with pollen costs $4.5 billion per year in the United States [41] and in a cost-benefit analysis of a dense automated system in Bavaria (Germany), Buters et al. argue that a 0.1% reduction in health costs for allergies alone could offset the cost of a denser automated network in that setting [25, 42]. Several U.S. states are exploring more robust pollen measurement networks [43], but solutions for funding challenges and integration of regional data need to be addressed. Successful implementation of pollen modeling for prediction will require concurrent developments in local pollen measurement, a better understanding of the role of the landcover factors, and improvements in atmospheric modeling.

While there are no documented examples of epidemic thunderstorms in the U.S., there is evidence that climate change is driving a lengthening pollen season with increasing pollen loads [8], and studies suggest that higher temperatures may lead to more frequent thunderstorms in the United States [44]. While the impact of U.S. thunderstorm asthma events is modest regarding emergency room visits and healthcare burden, better data collection would allow an improved exploration of this work and provide a better estimation of tools to predict these events that may worsen in the future. This study highlights the importance of additional pollen measurement in the United States for further research into this specific health outcome and other important research areas, including studies of allergenic response and the spatiotemporal spread of pollen.

## Supplementary information

Supplementary Information

## Data Availability

Health data is held by the Health Economics Program of the Minnesota Department of Health and is not publicly available. Pollen data was provided by the Clinical Research Institute of Minneapolis, Minnesota, and may not be shared by the authors. Weather data from Automated Surface Observation Stations is publicly available from the Iowa Mesonet [20], and lightning data is publicly available through the National Center for Environmental Information Severe Weather Data Inventory [17]. Please contact the authors with questions.
