# Supplementary material for: Can a single pollen measurement site provide exposure information for health research across an entire state? Results from a study of allergic-type asthma associated with thunderstorms (2007–2018)
Source: J Expo Sci Environ Epidemiol. 2025 May 5;36(1):143–8. doi: 10.1038/s41370-025-00777-z (PMC12795754; doi:10.1038/s41370-025-00777-z)
Supplement: Supplementary file 1 — Supplementary Information [file 41370_2025_777_MOESM1_ESM.docx]

**E Supplement Content** for ‘Can a single pollen measurement site provide exposure information for health research across an entire state? Results from a study of allergic-type asthma associated with thunderstorms’ by Smith ML, MacLehose RF, and Berman JD.

1. eTable 1:Meta-analysis of studies for all subgroups by age and sex.
2. eTable 2:Meta-Regression results of studies for the change in effect size for each additional 10 miles squared of deciduous tree, grass, or weed cover (RR(95% CI)) and I^2^ (%), and for change in effect size for each additional 10 miles of distance from MSP Airport), by age-sex subgroup.
3. eFigure1: The location of 19 study sites based on Core Based Statistical Area (CBSA) in Minnesota, with 15-mile radius for landcover shown. Zip codes partially or wholly within a 15-mile radius of the urban center (or of Minneapolis – St. Paul Airport for the MSP CBSA) are highlighted. Location of nearest Emergency Department, and nearest Automated Surface Observation Station also shown. The Minneapolis-St. Paul study area is labeled MSP.
4. eFigure2: Landcover types for Minnesota, 2013
5. eMethods: Expanded methods section describing exposure selection and parameterization, and models.

eTable 1:Meta-analysis of studies for all subgroups by age and sex, with number of included locations, results (RR of with confidence interval), and I^2^ for heterogeneity.

| Group | Included locations | Meta-analysis Results | I^2^ (%) |
| --- | --- | --- | --- |
| all population | 19 | 1.01 (0.96, 1.06) | 36.1 |
| all female | 19 | 1.03 (0.96, 1.09) | 37.7 |
| all male | 19 | 1.01 (0.96, 1.07) | 7 |
| all, U18 | 19 | 0.99 (0.92, 1.07) | 20.5 |
| all, 18-44 | 19 | 1.09 (1.04, 1.13) | 0 |
| all, 45 and up | 19 | 1.01 (0.95, 1.07) | 2.8 |
| male under 18 | 19 | 0.99 (0.93, 1.06) | 2.8 |
| male, 18-45 | 18 | 1.10 (1.03, 1.18) | 0.2 |
| male, 45 and up | 15 | 0.97 (0.88, 1.09) | 0 |
| female, under 18 | 18 | 1.03 (0.96, 1.11) | 0 |
| female, 18-45 | 19 | 1.08 (1.02, 1.13) | 0 |
| female, 45 up | 19 | 1.00 (0.91, 1.11) | 20.4 |
|  |  |  |  |

eTable 2:Meta-Regression results of studies for the change in effect size for each additional 10 miles squared of deciduous tree, grass, or weed cover (RR(95% CI)) and I2 (%), and for change in effect size for each additional 10 miles of distance from MSP Airport), by age-sex subgroup.

|  |  | Change in effect size per 10mile2 of deciduous tree, grass, or weed cover | | Change in effect size per mile of distance from MSP airport. | |
| --- | --- | --- | --- | --- | --- |
| Group | Included locations | RR (95% CI) | I^2^ (%) | RR (95% CI) | I^2^ (%) |
| all population | 19 | 1.009 (1.001, 1.017) | 24.3 | 0.992 (0.984, 1.000) | 22.4 |
| all female | 19 | 1.011(1.011, 1.021) | 16.8 | 0.992 (0.981, 1.003) | 29.7 |
| all male | 19 | 1.004 (0.993, 1.015) | 12.4 | 0.992 (0.984, 1.000) | 0.0 |
| all, U18 | 19 | 1.015 (1.001, 1.029) | 19.0 | 0.993 (0.985, 1.002) | 27.2 |
| all, 18-44 | 19 | 1.006 (0.995, 1.017) | 6.5 | 0.994 (0.984, 1.004) | 1.4 |
| all, 45 and up | 19 | 0.999(0.985, 1.013) | 5.2 | 0.992 (0.980, 1.004) | 0.0 |
| male under 18 | 19 | 1.009 (0.993, 1.026) | 11.7 | 0.992 (0.978, 1.008) | 0.5 |
| male, 18-45 | 18 | 1.006 (0.988, 1.024) | 6.6 | 0.989 (0.977, 1.002) | 0.0 |
| male, 45 and up | 15 | 0.990 (0.965, 1.016) | 0.0 | 0.994 (0.976, 1.013) | 0.0 |
| female, under 18 | 18 | 1.019 (1.002,1.037) | 0.0 | 1.001 (0.987, 1.015) | 0.0 |
| female, 18-45 | 19 | 1.009 (0.997, 1.022) | 0.0 | 0.994 (0.984, 1.005) | 4.1 |
| female, 45 up | 19 | 1.002 (0.983, 1.020) | 23.2 | 0.992 (0.976, 1.009) | 14.1 |

eFigure 1: The location of 19 study sites based on Core Based Statistical Area (CBSA) in Minnesota, with 15-mile radius for landcover shown. Zip codes partially or wholly within a 15-mile radius of the urban center (or of Minneapolis – St. Paul Airport for the MSP CBSA) are highlighted. Location of nearest Emergency Department, and nearest Automated Surface Observation Station also shown. The Minneapolis-St. Paul study area is labeled MSP.


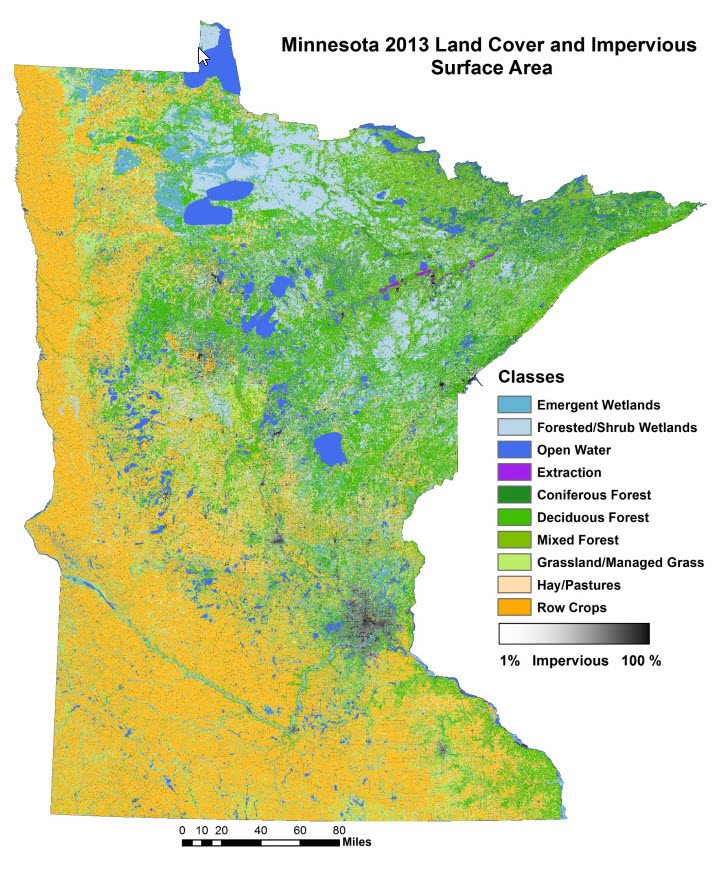


eFigure 2. Landcover types for Minnesota, 2013^1^

Expanded Methods Section:

**eMethods:**

*Study Population and Health Data*

We identified 24 potential urban study areas in Minnesota, US, based on the 24 US Census designated core-based statistical areas (CBSA) with urban centers of at least 10,000 people in the state of MN. Each study area included all zip code populations wholly or partially within 15 miles of the urban center of the CBSA,^2^ or the Minneapolis-St. Paul (MSP) airport for the Minneapolis/St. Paul/Bloomington metropolitan area. Five locations were excluded; 3 were removed because of overlapping boundaries and two because of lack of data. The final data set had 19 study areas with adequate sample sizes to converge in individual regression models. Daily total counts of asthma-related emergency department (ED) admissions as first or second ICD-9 diagnosis code of 493 or ICD-10 code of J45^3^ for all study area zip codes were estimated using Hospital Uniform Billing Claims Data collected by the Minnesota Hospital Association and accessed under agreement with the Health Economics Program of the Minnesota Department of Health (MDH).^4^ To investigate different age-sex responses, stratified data sets were created by sex of patient (male/female), age (under 18, 18-44, and 45 years and up), and age-sex combinations (under 18 male, under 18 female, 18-44 male, 18-44 female, 45 and up male, 45 and up female). Data was only accessible through the MDH secure network.

*Thunderstorm Asthma Events*

Thunderstorm asthma events are defined as two or more lightning strikes in a day occurring in the presence of high or very high pollen (>75^th^ percentile).^5^ This cut-off was based on National Allergy Bureau criteria and selected to allow comparison across various locations in future work.^5,6^ To assign lightning exposure, we first used a 0.1-degree grid of daily lightning counts from the VAISALA lightning detection network and created a smoothed grid of lightning exposure with inverse distance weighting.^7^ We assigned lightning counts based on the study centroid value of an inverse distance weighted interpolation of daily lightning count values collected at a 0.1-degree grid. Minnesota pollen data was available from the National Allergy Bureau at a single anonymous residential site in Minneapolis near the airport. Data is collected by certified pollen counters employed by the Clinical Research Institute of Minneapolis.^8^

*Environmental Data*

Landcover categories were obtained from a 15m resolution raster of the 2013 Minnesota Landcover Classification^1^ shown in eFigure 2. Using GIS tools, we assigned the total in square meters of each of the ten unique land cover types to each study area radius. Daily precipitation, maximum wind speed, average humidity, and maximum temperature were downloaded from the Iowa Mesonet system.^9^ Air pollution exposure data for daily maximum ozone and fine particulate matter (PM_2.5_) was assigned using an inverse distance weight of daily monitor values from the Environmental Protection.^10^ All weather and pollutant measures were assigned to the study area based on the value at Minnesota surface weather observation stations (AWOS or AWOS) serving as the center of each study area.

*Statistical Analysis*

Statistical analysis for each location builds on previous definition exploring the thunderstorm asthma phenomenon.^5^ For the whole population and each age-sex subgroup combination, we first ran a quasiPoisson regression model with day as the unit of analysis at each CBSA location for the 2007-2018 study period using all months from April to October. We calculated the rate of asthma ED visits associated with thunderstorm asthma events. A rate ratio was estimated from the covariate adjusted model. Separate models were fit for the effect of thunderstorm asthma events and asthma ED visits on the same day and with ED visits on the subsequent day (lag 1). It has been previously shown that thunderstorm asthma is an acute occurrence without substantial lagged effects beyond day one.^11^ Missing values for pollen data are estimated as the mean of the days that bound the missing period for three or fewer.^11^ The full model takes the form of equation [1]:

$\log$[E($y_{t}$)]$= B_{0} + B_{1}{event}_{t}{+B_{2}{Tmax}_{t} + B}_{3}{PM25}_{t} +B_{4}{Wind}_{t}+B_{5}{O3}_{t}+B_{6}{RH}_{t}+B_{7}{Precip}_{t}+B_{8}{dow}_{t} +offset+g(t)$ [1].

The outcome is daily counts of expected asthma ED visits, ${y,}$ on day *t*. *Event* on day t is the primary exposure of 2 lightning strikes in the presence of high or very high pollen, *Tmax* is the daily maximum temperature in degrees C, *PM25* is a value for daily mean PM_2.5_ (ug/m^3^), *Wind* is daily maximum wind speed, *O3* is the daily ozone maximum (ppb), *RH* is daily maximum relative humidity, *Precip* is daily precipitation total (mm), *DOW* is a categorical term for day of the week, and *g(t)* is a 6-knot cubic-spline term to control for seasonal trends in asthma ED visits. Knots per spline for the April – October annual period was chosen based on prior work in severe asthma ED visits using as few as one spline every two months ^12^ or as many as one spline each month.^13^ We use 5 year ACS surveys at the zip level and combine using the 2007-2011 for the year 2009, 2010-2014 for the year 2012, and 2013-2017 for 2015 and ran a simple linear regression. (R2 was near 100%). Then we assigned each intervening year the corresponding value based on this, and summed the zips for each CBSA.^14^

Sensitivity tests were conducted at 5 and 7 degrees of freedom. We also explored robustness of our thunderstorm asthma definition by using ‘anti’ exposures of pollen only (high or very high pollen in the absence of lightning) and lightning only (with pollen less than 25%).

In a second stage, we combined the individual study area results using random effects meta-analysis to calculate an overall estimate. Each study area-specific effect was weighted proportionally to the inverse of its variance, and the standard DerSimonian and Laird approach was used to estimate the random effect term.^61,62^ This analysis was repeated for subgroups, created based on age and sex, with analyses for the entire population, male, female, under age 18, 18-44, and 45 and up, and age-sex categories for male and female under 18, male and females 18-44, and male and female 45 and up. Categories were chosen to ensure adequate cell count, to identify children at risk, and to match other literature about severe asthma risks by age.

Following our meta-analysis, we fit a series of meta-regressions to investigate explanatory variables for between-site effects and investigate the ability to improve estimations over a large study area where pollen measures are absent through landcover surrogates. Our covariates included plant cover type, an overall landcover metric indicative of pollen, and measures of absolute distance from each site from the true pollen measurement location.

To estimate the effect of plant cover types, we used GIS tools to calculate the square miles of different plant types in each of our study areas for major land categories of conifer forest, deciduous forest, emergent wetland, extraction, forested and shrub wetland, hay and pasture, managed grass/natural grass, mixed forest, open water, and impervious (urban). To approximate overall pollen exposure representing the grass, tree, and weed pollen of health concerns, we created a composite value based on the square kilometers of total deciduous tree or managed grass/natural grass in each study region. Prior research indicates that grass, ragweed, birch, elm, hazel, and alder trees have all been associated with the distribution of sub-pollen particles in the specific conditions of a thunderstorm^15–18^, but not pines, which take up to 3 days^19^ to rupture into respirable sub pollen particles. We also calculated the percent of each study area covered by these plant type categories and the overall pollen exposure. Additionally, we calculated the distance^20^ from the centroid of each CBSA study unit to MSP airport, our proxy for our anonymized pollen collection center, along with the degrees of absolute latitude distance and absolute longitude distance. We fit meta-regression models on the whole population using these measures individually and, as a sensitivity analysis, re-ran these meta-regressions excluding Minneapolis – St. Paul. To test whether distance and landcover are associated, we measured the correlation between distance and square miles of deciduous trees or managed or natural grassland.

For sensitivity testing, first we ran a meta-analysis of models using an anti-exposure of lightning only (2+ strikes) in the presence of low pollen (less than 25^th^ percentile). Second, we ran separate meta-regressions to test whether any heterogeneity between lightning-only and asthma was modified by square miles of landcover hypothesized to contain involved pollen types or distance from MSP. Third repeated this process, first running a meta-analysis of models using high pollen as the exposure with no presence of a thunderstorm (less than two lightning strikes) and meta-regression using square miles of land cover with potential for asthma-producing pollen types and for distance from the MSP pollen collecting station. Fourth, we considered the effect of square miles of ‘all other land types’ as a counter to our meta-regression term of wild or managed grass and deciduous trees. Fifth, we tested a one-at-a-time addition of single landcover types to our pre-defined deciduous plus grass landcover measure.

For additional information about data and methods, please refer to the following manuscripts.

Smith ML, MacLehose RF, Chandler JW, Berman JD. Thunderstorms, Pollen, and Severe Asthma in a Midwestern, USA, Urban Environment, 2007–2018. *Epidemiology*. 2022;33(5). <https://journals.lww.com/epidem/fulltext/2022/09000/thunderstorms,_pollen,_and_severe_asthma_in_a.4.aspx>

Smith ML, MacLehose RF, Wendt CH, Berman JD. Sex and age characteristics of thunderstorm asthma emergency department visits. Hyg Environ Health Adv. 2024 Sep;11:100099. doi: 10.1016/j.heha.2024.100099. Epub 2024 Jul 17. PMID: 39391232; PMCID: PMC11466176.

Supplement Bibliography

1. Rampi LP, Knight JF, Bauer M. Minnesota Land Cover Classification and Impervious Surface Area by Landsat and Lidar: 2013-14 Update. Published online 2016.

2. Commons MG. City and Township Population Centers, MN.

3. Travers D, Lich KH, Lippmann SJ, et al. Defining Emergency Department Asthma Visits for Public Health Surveillance, North Carolina, 2008–2009. *Prev Chronic Dis*. 2014;11:130329. doi:10.5888/pcd11.130329

4. MDH. Asthma hospitalizations in Minnesota: facts & figures.

5. Smith ML, MacLehose RF, Chandler JW, Berman JD. Thunderstorms, Pollen, and Severe Asthma in a Midwestern, USA, Urban Environment, 2007–2018. *Epidemiology*. 2022;33(5). https://journals.lww.com/epidem/fulltext/2022/09000/thunderstorms,_pollen,_and_severe_asthma_in_a.4.aspx

6. American Academy of Allergy Asthma And Immunology; National Allergy Bureau. 2023. https://pollen.aaaai.org/#/

7. Vaisala. Vaisala’s NLDN US National Lightning Detection Network. Published online 2015.

8. Clinical Research Institute NAB pollen Counting Site. Pollen Data. 2021. Accessed December 19, 2024. https://www.criminnesota.com/

9. MESONET. Iowa Environmental Mesonet. Department of Agronomy, Univeristy of Iowa. 2019. Accessed December 7, 2024. https://mesonet.agron.iastate.edu/request/download.phtml?network=MN_ASOS

10. US Environmental Protection Agency. Air Quality System Data Mart. [internet database]. Accessed June 26, 2021. https://www.epa.gov/airdata.

11. Smith ML, MacLehose RF, Chandler JW, Berman JD. Thunderstorms, Pollen and Severe Asthma in a Midwestern, USA Urban environment, 2007-2018. *Epidemiology*. 2022;(Forthcoming).

12. Silverman RA, Ito K. Age-related association of fine particles and ozone with severe acute asthma in New York City. *Journal of Allergy and Clinical Immunology*. 2010;125(2):367-373.e5. doi:https://doi.org/10.1016/j.jaci.2009.10.061

13. Sinclair AH, Tolsma D. Associations and Lags between Air Pollution and Acute Respiratory Visits in an Ambulatory Care Setting: 25-Month Results from the Aerosol Research and Inhalation Epidemiological Study. *J Air Waste Manage Assoc*. 2004;54:1212-1218.

14. Manson S, Schroeder J, Riper D Van, Ruggles S. *IPUMS National Historical Geographic Information System: Version 13.0 [Database]*.; 2018. doi:http://doi.org/10.18128/D050.V13.0

15. Suphioglu C, Singh MB, Taylor P, et al. Mechanism of grass-pollen-induced asthma. *The Lancet*. 1992;339(8793):569-572. doi:10.1016/0140-6736(92)90864-Y

16. Grote M, Vrtala S, Niederberger V, Valenta R, Reichelt R. Expulsion of allergen-containing materials from hydrated rye grass (Lolium perenne) pollen revealed by using immunogold field emission scanning and transmission electron microscopy. *J Allergy Clin Immunol*. 2000;105(6 Pt 1):1140-1145.

17. Taylor PE, Flagan RC, Valenta R, Glovsky MM. Release of allergens as respirable aerosols: A link between grass pollen and asthma. *Journal of Allergy and Clinical Immunology*. 2002;109(1):51-56. doi:10.1067/mai.2002.120759

18. Miguel AG, Taylor PE, House J, Glovsky MM, Flagan RC. Meteorological Influences on Respirable Fragment Release from Chinese Elm Pollen. *Aerosol Science and Technologyfile:///Users/morrisonlukesmith/Downloads/short-term-exposure-to-pollen-and-the-risk-of-allergic-and-asthmatic-manifestations_-a-systematic-review-and-meta-analysis.ris*. 2006;40(9):690-696. doi:10.1080/02786820600798869

19. Taylor PE, Jacobson KW, House JM, Glovsky MM. Links between pollen, atopy and the asthma epidemic. *Int Arch Allergy Immunol*. 2007;144(2):162-170. doi:10.1159/000103230

20. Vincenty T. DIRECT AND INVERSE SOLUTIONS OF GEODESICS ON THE ELLIPSOID WITH APPLICATION OF NESTED EQUATIONS. *Survey Review*. 1975;23(176):88-93. doi:10.1179/sre.1975.23.176.88
